# Editorial: New approaches to overcome the blood-brain barrier for the treatment of CNS disorders

**DOI:** 10.3389/fncel.2025.1730250

**Published:** 2025-11-10

**Authors:** Mikko Gynther, Gert Fricker, Sabrina Petralla

**Affiliations:** 1 School of Pharmacy, University of Eastern Finland, Kuopio, Finland; 2 Institute of Pharmacy and Molecular Biotechnology, Heidelberg University, Heidelberg, Germany

**Keywords:** blood-brain barrier, CNS drug delivery, proton-coupled organic cation antiporter, immune response, nanoparticles, intranasal delivery

The primary obstacle to advancing successful treatments for neurodegenerative diseases is the blood-brain barrier (BBB) (Banks, 2016). Composed of polarized endothelial cells interconnected by tight junctions and several transporters, the BBB is a highly selective barrier known for its exceptionally low permeability, thereby restricting drug delivery to the Central Nervous System (CNS) (Pardridge, 2012). According to the World Health Organization (WHO) roughly 30% of the worldwide population is affected by neurological conditions (https://www.who.int/), and this prevalence is unfortunately expected to increase, causing progressively devastating social and economic impacts. Therefore, due to the lack of successful therapeutic strategies for CNS disorders, this Research Topic aimed to collect novel approaches and new perspectives on this issue.

Growing evidence has reported proton-coupled organic cationic antiport (H^+^/OC) as a valid strategy for CNS drug delivery, functionally expressed not only in the rat BBB cell line, but also in the immortalized human brain capillary endothelial cell line hCMEC/D3 (Sadiq et al., 2011; Shimomura et al., 2013). Indeed, numerous organic cation drugs utilized in clinical settings enter the brain across the BBB through this antiporter, indicating its potential for engineering CNS-active compounds with improved brain access (Doetsch et al., 2022; Bällgren et al., 2024; Debori et al., 2024; Svane et al., 2024).

The study by Bällgren et al. provides more insight into the proton-coupled organic cation (H^+^/OC) antiporter system, investigating the pharmacokinetics of six substrates—pyrilamine, diphenhydramine, bupropion, tramadol, oxycodone, and memantine—in male Sprague-Dawley rats. Using the Combinatory Mapping Approach for Regions of Interest, the authors measured unbound drug concentrations in brain, BBB, blood–spinal cord barrier (BSCB), and blood–cerebrospinal fluid barrier (BCSFB). All drugs showed active uptake across the BBB and BSCB, with unbound brain-to-plasma concentration ratios (Kp,uu,ROI) consistently above one. No differences in uptake were observed between whole brain vs. individual brain regions, indicating a region-independent transport. The analysis also revealed intracellular accumulation of the drugs, likely via lysosomal trapping, which may contribute to high tissue retention and possibly neurotoxicity.

Neurodegenerative disorders, as well as ischemic stroke, lead to an impairment of BBB with its transient opening which allows peripheral immune cells, including T cells, to infiltrate the brain parenchyma (Li et al., 2018; Qiu et al., 2021). Therefore, understanding and locally modulating the behavior of T cells may represent a novel approach in stroke research. The study by Telec et al. aims to refine our understanding of the peripheral T cell landscape after an ischemic stroke, thereby establishing the basis for designing cell-specific delivery strategies to modulate neuroimmune interactions. The colleagues measured subgroups of circulating T-cell, including CD4^+^, CD8^+^, and double-negative CD4^+^CD8^+^ T cells, in patients with acute ischemic stroke compared to control subjects at different time points. No significant differences were observed in the absolute number or proportions of these T cell subgroups between stroke patients and controls. However, higher percentages of CD4^+^ T cells and CD4^+^/CD8^+^ ratio correlated with better neurological status in the acute and subacute phases. In contrast, the proportions of CD8^+^ were inversely related. Therefore, the balance between CD4^+^ and CD8^+^ could reflect the host's immune status after a stroke and be related to prognosis.

Recent progress in nanotechnology has provided the development of novel platforms to deliver therapeutic agents across the BBB (Toader et al., 2024; Rafieezadeh, 2025). Among them, Li et al., described ROS-responsive GO@GSH-FA nanoparticles as a therapeutic strategy for mitigating cerebral ischemia-reperfusion (I/R) injury. They developed a graphene oxide (GO)—based nanocomposite, named GO@GSHFA, which selectively releases the antioxidant glutathione (GSH) into ischemic brain tissue during cerebral I/R damage. The authors included a fibrinogen-binding aptamer (“FA”) in the GO so that the nanocomposite preferentially hosts fibrin-rich thrombotic regions in the injured brain. Moreover, the GSH is conjugated via a ROS-sensitive borate ester linker, allowing for cleavage of the linker in zones of high oxidative stress, thereby releasing the GSH locally.

GO@GSHFA was tested *in vitro*, restoring neuronal cell viability (SH SY5Y) under oxygen and glucose deprivation/reperfusion conditions, reducing ROS and inflammatory cytokines, and decreasing apoptosis. In addition, in a mouse model of cortical ischemia, intravenous GO@GSHFA administration accumulated preferably in ischemic brain tissue, improving neurological deficits and preserving neuronal survival compared to untreated controls. Overall, the nanoplatform described has the potential to enhance both site specificity and the therapeutic index for delivering neuroprotective agents across the BBB in stroke scenarios.

Among the alternative administrative routes, intranasal delivery is certainly one of the most promising and non-invasive approaches to target neurodegenerative disorders due to its rapid absorption and ability to bypass the BBB (Correale et al., 2025; Majie et al., 2026).

In the last article of this Research Topic by Disdier et al., the authors studied dodecyl creatine ester (DCE), a modified prodrug of creatine, also called CBT101, delivered intranasally to a rat model affected by Parkinson's disease (PD) with unilateral 6-hydroxidepamine (6 OHDA) lesions. Following intranasal administration of DCE for 5 weeks, rats showed improved motor symmetry and sensorimotor performance compared to vehicle controls in the behavioral tests of amphetamine-induced rotation and beam walking. In addition, treated animals had increased levels of striatal dopamine in the injured hemisphere compared to vehicle, along with modulation of BDNF/pro BDNF balance and partial rescue of neurofilament proteins in striatum and plasma.

Therefore, this study represents a concrete example of how a prodrug in concomitance with intranasal route can overcome BBB-related administration bottlenecks for CNS therapies.

In conclusion, the contributions featured in this edition explore advanced and non-invasive strategies—such as intranasal delivery of peptides, small molecules, and nanoparticles—for successfully overcoming the BBB. This increasing body of work points to positive directions for improving therapeutic availability to the brain. Continued advancements in this field may ultimately revolutionize therapeutic approaches for neurological disorders.
